# Genetic Characterization of *Plasmodium falciparum* Histidine-Rich Protein 2 Deletions and Their Impact on Malaria Interventions in Odisha, India

**DOI:** 10.4269/ajtmh.24-0330

**Published:** 2024-12-17

**Authors:** Stuti Mohanty, Abbey M. Jones, Swagatika Dash, Satya Ranjan Chhatria, Timir Kanta Padhan, Sanjib Mohanty, Jane M. Carlton, Danielle C. Ompad, Anne Kessler, Praveen Kishore Sahu

**Affiliations:** ^1^ Department of Molecular Biology and Infectious Diseases, Community Welfare Society Hospital, Rourkela, Odisha, India;; ^2^ Department of Epidemiology, School of Global Public Health, New York University, New York, New York;; ^3^ Center for Genomics and Systems Biology, Department of Biology, New York University, New York, New York;; ^4^ Johns Hopkins Malaria Research Institute, Department of Molecular Microbiology and Immunology, Johns Hopkins Bloomberg School of Public Health, Baltimore, Maryland

## Abstract

Diagnostic escape via *Plasmodium falciparum* (*P. falciparum*) histidine-rich protein 2 (*pfhrp2*) gene deletions is a major potential hurdle for global malaria elimination efforts. We investigated the prevalence of *pfhrp2* gene deletions in 15 malaria-endemic villages in the state of Odisha, India, and modeled their impact on an ongoing in-country malaria intervention program. We found that 61.6% of subpatent *P. falciparum* infections (i.e., rapid diagnostic test [RDT]-negative and positive by polymerase chain reaction [PCR]) had *pfhrp2* gene deletions, which were predominantly located in the exon 2 region (96.2%) and largely identified in samples from febrile individuals (82.6%). DNA sequencing and protein diversity features were characterized in a subset of samples from individuals with subpatent infections carrying intact *pfhrp2* exon 2 loci. Our analyses revealed novel amino acid repeat motifs (231–293 amino acids), and these variant repeat sequences differed from those of RDT+/PCR+ samples. We also evaluated the state-sponsored mass screening and treatment intervention in the context of *pfhrp2* gene deletions. We found that mass screening and treatment conducted alongside additional interventions (e.g., long-lasting insecticidal net distribution, indoor residual spraying) reduced the relative risk of infection for both *P. falciparum* parasites harboring a *pfhrp2* deletion (adjusted relative risk ratio [aRRR] = 0.3; 95% CI = 0.1–1.0) and *P. falciparum* parasites with intact *pfhrp2* genes (aRRR = 0.4; 95% CI = 0.2–1.1) when compared with the use of mass screening and treatment by RDT alone. Combined, our findings highlight the need for alternative diagnostic targets and tools as India moves toward its goal of malaria elimination by 2030.

## INTRODUCTION

Malaria continues to be a major agent of morbidity and mortality globally, especially in tropical and subtropical regions.^1^ The WHO reported an estimated 249 million malaria cases and 608,000 malaria deaths occurring worldwide in 2022, with India carrying the largest proportion of infections (66%) in the Southeast Asia region, with an estimated 3.38 million cases.^1^ Among the five *Plasmodium* species responsible for infections in humans, *Plasmodium falciparum* (*P. falciparum*) confers the highest burden of human morbidity and mortality.^1^ The rapid diagnosis of infection, followed by prompt and appropriate treatment, is the cornerstone of malaria control in terms of case management and the interruption of transmission.^2^

*Plasmodium* infections should be confirmed using diagnostic testing before administering antimalarial treatment.^2^ Microscopy has long been the gold standard for malaria diagnosis in low-resource settings, including rural India, as it is the most cost-effective option and allows for the direct detection of parasite density and stage by species.^3^ The evaluation of stained smears using light microscopy, however, requires technical expertise and the thorough maintenance of infrastructure, with proper quality control.^4^ Given the short timeline to diagnosis and the absence of need for additional equipment or expertise, rapid diagnostic tests (RDTs) have become an increasingly used diagnostic method.^5^ Commercially available RDTs are designed to detect parasite-specific proteins or antigens, namely, species-specific or pan-*Plasmodium* lactate dehydrogenase, pan-*Plasmodium* aldolase, or *P. falciparum*-specific histidine-rich protein 2 (PfHRP2).^6^ Among the RDTs available for *P. falciparum* infection detection, those targeting PfHRP2 are most widely used for several reasons, including the abundant expression of the target protein during the asexual stages of the parasite’s life cycle,^7^ the target protein’s structural stability^8^ and capability of being recognized by more than one antibody.^9^ More than 415 million *Plasmodium* RDTs were sold worldwide in 2022, the majority of which detected PfHRP2.^1^

*Plasmodium falciparum*-specific histidine-rich protein 2 is encoded by a single copy gene located in the sub-telomeric region of *P. falciparum* chromosome eight (Supplemental Figure 1A) and consists of two exons and one intron.^10^ Full detection of the *pfhrp2* gene is achieved through amplification of the following regions: 1) the entirety of exon 2; 2) the region spanning exon 1–2; and 3) two flanking genes (i.e., the upstream and downstream regions).^11^ Deletions occur in large part because of breakage and rejoining at the unstable subtelomeric region where the gene is located.^12^ The *pfhrp2* gene is structurally similar to *pfhrp3* gene, which may be detected by RDTs targeting PfHRP2.^9^ This cross-reactivity is more likely to occur in instances of high parasitemia;^12^^–^^15^ however, false negative RDT results have also been widely documented in infections with high parasitemia, and in many settings, co-occurring with *pfhrp2* and *pfhrp3* deletions.^16^^–^^19^

False negative results (i.e., diagnostic escape) of *P. falciparum* infections from RDTs targeting PfHRP2 can occur because of low parasitemia, prozone effect, the quality and handling of diagnostic test kits, and full or partial deletions in the *pfhrp2* gene.^20^ The emergence of partial or complete deletions in the *pfhrp2* gene is an increasingly serious threat to malaria elimination goals globally.^1^ A recent evaluation of malaria RDTs found 85 tests for *P. falciparum* infection that met the WHO’s performance criteria.^21^ Of these, 83 detected the antigen PfHRP2.^21^ There are currently no WHO-prequalified RDTs that distinguish between *P. falciparum* and *Plasmodium vivax* (*P. vivax*) without detecting HRP2.^1^^,^^15^ The widespread and global use of PfHRP2-based RDTs, coupled with the emergence of *pfhrp2* deletions in the *P. falciparum* parasite, has led to concern regarding the rising rate of RDT failure.^1^

Deletion in the *pfhrp2* and *pfhrp3* genes was first detected in retrospectively analyzed field isolates collected in 2007 from Peru,^22^ and further genetic analyses of neutral microsatellite markers around the *pfhrp2* gene revealed the presence of different haplotypes, indicating that deletion was not a single event and had likely occurred several times in Peru.^23^ Subsequently, *pfhrp2*/3 gene deletions were reported in other countries throughout South America, Africa, and Asia.^17^^–^^19^^,^^24^^,^^25^ In India, *pfhrp2* and *pfhrp3* gene deletions were first detected in field isolates from Chhattisgarh state in central India.^8^ Previous studies conducted in Odisha State, which carried the highest malaria burden in India in 2020,^26^ identified *pfhrp2* deletion harboring parasites in samples collected between 2013 and 2016.^27^^,^^28^

India has made substantial progress in malaria control over the past decade through various strategies, including streamlined program management, routine active case surveillance, targeted intervention strategies, and improved early access to malaria diagnosis and treatment.^29^ In 2016, the Odisha State malaria control program deployed a multi-component “malaria camp” (MC) intervention through a statewide malaria elimination initiative called Durgama Anchalare Malaria Nirakaran (DAMaN), which in local language means malaria elimination in remote and inaccessible areas. The MC intervention included mass screening and treatment using RDT, the distribution of long-lasting insecticidal nets, indoor residual spraying and other vector control methods, and educational programming to target highly malaria-burdened communities within the state.^30^^,^^31^ An 88% decrease in the malaria burden from 2017 to 2018 was reported by the state after the initial application of the intervention.^30^^,^^32^ Our recent evaluation of the intervention found that the MCs were significantly associated with a reduction in malaria prevalence and were measured as *Plasmodium* infections detected using polymerase chain reaction (PCR).^33^ However, because the primary tests used for this program are PfHRP2-detecting RDTs, further information is needed on the differential effects of the multicomponent intervention on *P. falciparum* infections with and without *pfhrp2* gene deletions.

Understanding whether control efforts and large-scale interventions will be effective at reducing infections with *pfhrp2* deletions is important for developing and tailoring ongoing and new strategies targeting elimination. Overall, information on the prevalence of *pfhrp2* gene deletions in India remains sparse.^8^^,^^27^^,^^28^ The multidisciplinary study described here was undertaken to assess the prevalence of *pfhrp2* gene deletions in the malaria-endemic remote villages of Odisha State, India, as well as to examine the impact of these gene deletions on the ongoing malaria intervention program in the region.

## MATERIALS AND METHODS

### Ethics statement.

This study used a subset of preexisting samples collected as part of an ongoing community-based study, wherein written informed consent was obtained from all participating adults and parents or legal guardians of minors.^31^^,^^33^ Ethics approval for the project was obtained on June 24, 2019 from the Research and Ethics Committee under the Directorate of Health Services, Odisha, and from the Institutional Ethics Committee at the Community Welfare Society (CWS) Hospital, Rourkela, Odisha, and the Institutional review board of New York University, New York, New York.

### Study sites and sample collection.

Details of the study sites, study population, and sample collection procedures are described elsewhere.^31^^,^^33^ Briefly, the study was conducted between August 2019 and November 2020 within a quasi-experimental, cluster-assigned framework designed to evaluate the effectiveness of the DAMaN MC intervention in reducing the prevalence of *Plasmodium* infection.^33^ Fifteen villages in the districts of Jharsuguda and Keonjhar in Odisha, India, were nonrandomly assigned to three study arms. Villages in arm A (*n* = 6) were assigned to newly receive the MC intervention during the study period. Villages in arm B (*n* = 6) were assigned to a delayed start of the MC intervention and served as the comparison condition during the study period. A third set of villages (*n* = 3), arm C, had begun receiving the MC intervention before the beginning of the study. The enrollment of study participants occurred concurrently with the initiation of the MC intervention in arm A. All study participants (*N* = 2,463) were scheduled to attend four study visits between August 2019 and November 2020; samples were collected from consenting participants at each study visit. Samples were also collected from malaria patients visiting the outpatient department of CWS Hospital in Rourkela, Odisha. All assays were performed on finger prick blood specimens collected from individuals aged 1–69 years after obtaining their written informed consent. Participants with a body temperature of ≥99.5°F were considered febrile.

### Enrollment and collection of clinical control sample set.

Finger prick blood samples and history of fever were obtained from febrile patients (*n* = 10) presenting to the outpatient department at CWS Hospital in Rourkela, Odisha, India, between September 2019 and November 2021 after obtaining written informed consent (and assent, when appropriate). All participants tested positive for *P. falciparum* infection with PfHRP2-based RDTs, and the fingerprick blood samples were collected before each malaria patient received antimalaria treatment according to the national antimalarial drug policy of India.^34^

### *Plasmodium* infection detection by RDT.

Blood samples collected by sterile finger prick from each study participant at each study visit were tested in the field for the presence of *P. falciparum* or *P. vivax* parasites using a bivalent RDT (Falcivax, Zephyr Biomedicals, Goa, India) targeting the PfHRP2 antigen and the *P. vivax* lactate dehydrogenase (PvLDH) antigen.

### DNA isolation and confirmation of *Plasmodium* infection by PCR.

DNA was isolated from 200 µL of packed red cells obtained from finger prick samples using the QIAamp DNA mini kit (Qiagen, Hilden, Germany). Total DNA was eluted in 50 *µ*L aliquots and stored at −20°C until assayed. *P. falciparum* and *P. vivax* infections were confirmed by using species-specific PCR (ssPCR) assays, as previously described,^31^ using primers targeting multicopy loci Pvr47 (*P. vivax*) and Pfr364 (*P. falciparum*).^35^

### Amplification of the *pfhrp2* gene and its flanking genes.

DNA samples isolated from subpatent *P. falciparum* infections (i.e., negative by RDT and positive by PCR) were assayed in a multi-PCR workflow for the presence of deletions in the *pfhrp2* genetic region, as previously described.^27^ Briefly, samples were first subjected to PCR amplification of *pfhrp2* exon 2, and those that amplified were subsequently used in an amplification reaction for the *pfhrp2* exon 1–2 region. Samples with an intact exon 1–2 region were characterized for the presence of intact flanking genes, MAL7P1.230 (upstream) and MAL7P1.228 (downstream). Positive (*P. falciparum* strain 3D7) and negative (*P. falciparum* Dd2 strain) controls were used for all PCRs in the workflow, as were clinical malaria control samples (PfHRP2 RDT+ and ssPCR+). Approximately 10% (*n* = 246) of samples were tested at an independent facility for quality control.

### Quantitative PCR assay targeting the *Pf aldolase* gene.

Samples that failed to amplify any region of the *pfhrp2* gene targeted in the multi-assay workflow were subjected to quantitative PCR (qPCR) targeting the *P. falciparum aldolase* gene to confirm the quality and integrity of the DNA sample.^36^ The cycle threshold (Ct) cutoff was set at less than 35 cycles, and samples with Cts of >35 or <11 were deemed inconclusive. The assay controls were as described for the *pfhrp2* PCR assay.

### Estimation of PfHRP2 in plasma samples by ELISA.

PfHRP2 was detected and quantified in a subset of plasma samples (*n* = 17) obtained from study participants with subpatent *P. falciparum* infections and control samples (*n* = 9) obtained from individuals presenting to the CWS Hospital’s outpatient clinic who were positive for *P. falciparum* infection by both RDT and PCR, with intact *pfhrp2* genes. A commercially available ELISA kit **(**Malaria Ag CELISA kit, Cellabs, Sydney, Australia) was used according to the manufacturer’s instructions, absorbances were read at 450 nm using a Multiskan Spectrum ELISA plate reader (ThermoFisher, Waltham, MA), and the values were estimated in ng/ml by using GraphPad Prism (v 10.2.0, GraphPad Prism, Boston, MA).

### Determination of *P. falciparum* parasitemia by light microscopy.

Light microscopy of Giemsa-stained thin blood smears was performed to estimate peripheral *P. falciparum* parasitemia in a subset (*n* = 8) of the clinical control samples and RDT–/ssPCR+ samples for which a blood smear was available. Briefly, asexual *P. falciparum* parasite forms were counted against 200 leukocytes (i.e., number of parasites per 200 white blood cells [WBCs]). Total parasitemia per µL of blood was subsequently calculated by multiplying the total WBC counts by 8,000.

### DNA sequencing, editing, and protein characterization.

A subset of field samples (*n* = 20) from individuals with subpatent *P. falciparum* infections (i.e., RDT-negative and PCR-positive) and control samples (*n* = 10) obtained from individuals presenting to the clinic who were positive for *P. falciparum* infection by both RDT and PCR with intact *pfhrp2* exon 2 regions were amplified (∼814 bp) and sequenced by using Sanger sequencing in both forward and reverse directions on an ABI3730xl sequencer (Applied Biosystems, Waltham, MA) at Heredity Life Sciences (Bhubaneswar, Odisha). The sequencing chromatograms were reviewed manually for base calling errors by using FinchTV (version 1.4, Geospiza Inc, Denver, CO). The forward and reverse sequences of each sample were collated to obtain a single sequence using Lasergene 17’s SeqBuilder module (DNASTAR, Madison, WI). All sequences were deposited in GenBank (GenBank accession nos. OM337920, OM337921, OM337922, OM337923, OM337924, OM337925, OM337926, OM337927, OM337928, OM337929, OM337930, OM337931, OM337932, OM337933, OM337934, OM337935, OM337936). Protein sequences were obtained by translating the contigs using Expasy (Swiss Institute of Bioinformatics, Lausanne, Switzerland).^37^ Repeats in the amino acid sequences were identified manually across the exon 2 region and assigned previously published numeric codes (Supplemental Figure 1A).^12^^,^^38^ The multiplication product of the quantities of type 2 and type 7 repeats (type 2 × type 7) was used to group the samples into four groups^38^ as follows: group A (very sensitive), when the product was greater than 100; group B (sensitive), when the product was between 50 and 100; group I (borderline), when the product was between 44 and 49; and group C (non-sensitive), when the product was less than 43 (Supplemental Table 1).

### Analysis of the intervention’s impact on infections with and without a *pfhrp2* deletion.

Data collected at baseline and at the third follow-up visit for all participants in study arms A and B were included. The outcome of interest, *pfhrp2* deletion status, was categorized into three levels: 0) no *Plasmodium* infection detected by PCR or RDT; 1) RDT–/PCR+ *P. falciparum* infection with laboratory-confirmed *pfhrp2* deletion; and 2) *P. falciparum* infections with assumed intact *pfhrp2* gene (either RDT+ *P. falciparum* infection or RDT–/PCR+ *P. falciparum* infection with laboratory-confirmed presence of *pfhrp2* gene). Any *Plasmodium* infection that did not meet the criteria for outcome category 1 or 2 as described was considered missing for this analysis.

Bivariable associations between covariates and *pfhrp2* deletion status at baseline were examined by using Pearson’s χ^2^ or Fisher’s exact tests. Intervention effects were estimated using multilevel, multinomial logistic regression. An interaction term of study arm and study visit was included to estimate intervention effects at the third follow-up visit. Relative risk ratios (RRRs) and 95% CIs were calculated for each infection category compared with no infection. The RRR and 95% CI for *pfhrp2* deletion infections compared with intact *pfhrp2* infections were also calculated. Estimates adjusted for participant sex and age were calculated. Models accounted for the clustering of observations within individuals within villages. Analyses were conducted by using Stata 17.0 (StataCorp, College Station, TX).

## RESULTS

### *Plasmodium falciparum* infections detected were largely subpatent.

A total of 6,662 blood samples were collected during the community study^33^ and tested for *Plasmodium spp.* infection by using a FalciVax RDT (Zephyr Biomedicals, Goa, India), of which 66 were positive for *P. falciparum*, and five were positive for *P. vivax* and one mixed infection (both *P. falciparum* and *P. vivax*). Molecular detection by PCR was also applied to all samples and confirmed 258 additional *P. falciparum* infections and three additional *P. vivax* infections, along with the infections already detected using RDT. Of the 258 subpatent *P. falciparum* infections, 115 were febrile and 143 were afebrile. Two RDT-positive samples for *P. falciparum* were found to be negative by using PCR.

### *pfhrp2* gene deletions were detected in the majority of subpatent *P. falciparum* infections, predominantly in the exon 2 region.

Deletions were detected in more than half of all subpatent *P. falciparum*-infected samples (61.6%; *n* = 159), and a higher proportion of febrile samples (82.6%; *n* = 95) had deletions in the *pfhrp2* gene than their afebrile counterparts (44.8%; *n* = 64; Table 1). Independent of fever status, most deletions (96.2%; *n* = 153) were detected in the exon 2 region, of which 94 were febrile and 59 were afebrile (Table 2). The remaining febrile samples had a deletion in the exon 1–2 region, and no deletions were detected in the upstream or downstream regions (Table 2). All five of the remaining afebrile samples had a deletion detected in the upstream flanking gene (Table 2). All regions of the *pfhrp2* gene were successfully amplified in the PfHRP2 RDT+ control samples (*n* = 10).

**Table 1:** *pfhrp2* gene deletion prevalence in subpatent (rapid diagnostic test-negative/polymerase chain reaction-positive) *Plasmodium falciparum* infections

Number of *P. falciparum* Infections	Total	Fever	No Fever
	258	115	143
*pfhrp2*-negative	159 (61.63)	95 (82.61)	64 (44.76)
*pfhrp2*-positive	74 (28.68)	04 (3.48)	70 (48.95)
Inconclusive	13 (5.04)	13 (11.30)	00 (0.00)
Not tested	12 (4.66)	03 (2.61)	09 (6.29)

*P. falciparum, Plasmodium falciparum; pfhrp2, Plasmodium falciparum* histidine-rich protein 2.

**Table 2:** Prevalence of *pfhrp2* gene deletions by targeted gene region and fever status

*pfhrp2* Target	Exon 2 Del +		Exon 1–2 Del +		UPS Del +		DWN Del +	
Fever status	Febrile (*n* = 95)	Afebrile (*n* = 64)	Febrile (*n* = 95)	Afebrile (*n* = 64)	Febrile (*n* = 95)	Afebrile (*n* = 64)	Febrile (*n* = 95)	Afebrile (*n* = 64)
No. of samples (%)	94 (98.9)	59 (92.2)	1 (1.1)	0 (0.0)	0 (0.0)	5 (7.8)	0 (0.0)	0 (0.0)

Del, deletion; DWN, downstream; *pfhrp2, Plasmodium falciparum* histidine-rich protein 2; UPS, upstream.

### qPCR of the housekeeping gene confirmed the *pfhrp2* gene deletion results.

Samples with a deletion detected in any region of the *pfhrp2* gene were assayed by using qPCR for the *P. falciparum aldolase* gene to confirm DNA integrity. A total of 172 samples yielded no amplification products for any of the *pfhrp2* targets. Of the 172 samples assayed, 159 were positive for the metabolic housekeeping gene, *P. falciparum aldolase*, and the remaining 13 samples did not amplify; therefore, they were deemed inconclusive (Table 1).^36^

### Characterization of the *pfhrp2* exon 2 region revealed novel repeats and inferred differences in RDT sensitivity.

A subset of PfHRP2 RDT–/ssPCR+ samples (*n* = 17) from the field sites with an intact *pfhrp2* exon 2 region were sequenced by Sanger sequencing in forward and reverse directions, and read lengths in the range of 726–890 bp were obtained. After translation of the sequences, 13 unique amino acid sequences were obtained from the 17 samples sequenced (Supplemental Figure 1B). Across all samples, the size of the *pfhrp2* exon 2 ranged from 231 to 293 amino acids. All 17 samples began with the type 1 repeat motif and ended with a type 12 repeat with different combinations of type 2, 3, 4, 5, 6, 7, 8, and 19 occurring in between (Supplemental Figure 1B). A variant of repeat type 7 and type 12 with amino acid substitutions at two positions were also detected in individual samples (Table 3). Among the clinical malaria control group (PfHRP2 RDT+) samples, the HRP2 protein was found to consist of 187–274 amino acids. Like the RDT–, PCR+ samples, the same repeat types were detected in this sample set, as well as two additional repeat types, 10 and 13 (Table 3; Supplemental Figure 1C). In the control samples, type 2 repeats were the most abundant. The variants of repeat types 2, 7, and 19 were detected in this set of samples, with repeat type 19 being novel in nature. Among the variants detected, there was only one common variant between the RDT– and RDT+ sample sets, a variant of type 2 repeats (Table 3).

**Table 3:** Variant protein sequences characterized in the *pfhrp2* exon 2 regions of rapid diagnostic test-negative/species-specific polymerase chain reaction-positive versus rapid diagnostic test-positive/species-specific polymerase chain reaction-positive samples

Repeat Type	Repeat Sequence	Variant Sequence in RDT-/ssPCR+ Samples	Variant Sequence in RDT+/ssPCR+ Samples
Type 1	AHHAHHVAD	–	–
Type 2*	AHHAHHAAD	AHHAHHA**D**D^†^	AHHAHHA**D**D^†^
			AHHAHHA**P**D^†^
Type 3	AHHAHHAAY	–	–
Type 4	AHH	–	–
Type 5	AHHAHHASD	–	–
Type 6	AHHATD	*APHATD* ^‡^	–
Type 7*	AHHAAD	AHHA**D**D^†^	AHHA**H**D^†^
		*A* ***P*** *HAAD* ^‡^	
Type 8*	AHHAAY	*A* ***P*** *HAAY* ^‡^	–
Type 12*	AHHAAAHHEAATH	*AH* ***P*** *AAAHHEAA* ***M*** *H* ^‡^	–
Type 19*	AHHAA	*A* ***P*** *HAA* ^‡^	AHHA**D**^‡^

*pfhrp2*, *Plasmodium falciparum* histidine-rich protein 2; RDT, rapid diagnostic test; ssPCR, species-specific polymerase chain reaction; –, No variants detected.

* Repeat types with variants detected, variant aa highlighted in bold.

^†^ Reported by Nderu et al., 2019.

^‡^ Detected in the present study. Italicized sequences indicate novel repeat variant sequences found in the subpatent field samples (RDT–/ssPCR+) with intact *pfhrp2* gene that were not found in the clinical control samples (RDT+/ssPCR+) with an intact *pfhrp2* gene.

The RDT sensitivity of the RDT–/ssPCR+ samples (*n* = 17) and the RDT+/ssPCR+ clinical control samples (*n* = 10), all with intact *pfhrp2* exon 2 regions, was predicted based on the multiplication product of the number of type 2 and type 7 repeats present, which allows for grouping into four categories A, B, I, and C.^38^ One (5.9%) RDT–/ssPCR+ sample was found to be in group A (very sensitive; Supplemental Table 1). Of the remaining RDT–/ssPCR+ samples, 10 (58.8%) were classified as group B (sensitive), two (11.8%) were classified as group I (borderline sensitive), and four (23.5%) were classified as group C (not sensitive). Out of the control samples (*n* = 10), three (30%) were found to be from group B (sensitive), two were from group I (borderline sensitive), and 5 (50%) were classified as group C (not sensitive).

### PfHRP2 levels and peripheral parasitemia were higher in plasma samples from clinical controls.

A comparison of the PfHRP2 antigen levels in the RDT–/ssPCR+ samples (*n* = 17) and nine of the RDT+/ssPCR+ control samples suggests higher antigen levels in the control samples, albeit a statistically insignificant comparison (Supplemental Figure 2A). A comparison of peripheral parasitemia as determined by light microscopy of stained blood smears in a subset of the same samples for which blood smears were available indicates lower parasitemia in the RDT–/ssPCR+ samples (Supplemental Figure 2B).

### Determination of the intervention’s impact on infections with and without a *pfhrp2* deletion.

An analysis was conducted to examine the differential impacts of the MC intervention on the prevalence of *P. falciparum* infections with and without a *pfhrp2* deletion. Participants in study arm A received the MC intervention during the study period, whereas participants in study arm B did not receive the MC intervention until after the study period concluded; however, they received mass screening by RDT due to study participation. Therefore, the comparison of these study arms examined the differences between receiving the complete MC intervention versus mass screening by RDT only. The analytic sample used for the evaluation of the intervention included 2024 participants from study arms A (*n* = 1,001) and B (*n* = 1,023). The third follow-up visit was completed for 80.7% of the study sample. Study participants with *P. falciparum* infections at baseline were younger than participants who were not infected (Table 4). Participants with infections that had an intact *pfhrp2* gene were more likely to be asymptomatic (82.9%) than those with infections with the *pfhrp2* gene deletion (27.1%; *P* <0.001).

**Table 4:** Baseline characteristics associated with *Plasmodium falciparum* infections with and without *pfhrp2* deletions in study arms A and B (*n* = 2,024)

Baseline Characteristics	Total, *N* (%), 2,024 (100.0)	Not Infected, *n* (%), 1,801 (100.0)	*P. falciparum* Infection With *pfhrp2* Deletion, *n* (%), 129 (100.0)	*P falciparum* Infection With Intact *pfhrp2*, *n* (%), 70 (100.0)	Missing/Inconclusive,* *n* (%), 24 (100.0)	*P*-value^†^
Study arm						
A (new MC)	1,001 (49.5)	860 (47.8)	93 (72.1)	38 (54.3)	10 (41.7)	<0.001
B (delayed MC)	1,023 (50.5)	941 (52.2)	36 (27.9)	32 (45.7)	14 (58.3)	
Sex						
Male	893 (44.2)	783 (43.5)	65 (50.4)	30 (43.5)	15 (62.5)	0.132
Female	1,128 (55.8)	1,016 (56.5)	64 (49.6)	39 (56.5)	9 (37.5)	
Missing	3	2	0	1	0	
Age in years						
Mean (SD, range)	26.4 (19.3, 1–69)	27.0 (19.4, 1–69)	22.3 (18.5, 1–68)	20.8 (17.7, 1–65)	21.0 (18.8, 1–60)	0.002^‡^
Age in years (categorical)						
5 and under	273 (13.5)	234 (13.0)	24 (18.6)	10 (14.3)	5 (20.8)	0.009
6 to 17	563 (27.8)	480 (26.7)	44 (34.1)	30 (42.9)	9 (37.5)	
18 to 34	486 (24.0)	442 (24.5)	27 (20.9)	14 (20.0)	3 (12.5)	
35 and over	702 (34.7)	645 (35.8)	34 (26.4)	16 (22.9)	7 (29.2)	
Educational attainment						
No school/less than primary	996 (49.2)	869 (48.3)	68 (52.7)	45 (64.3)	14 (58.3)	<0.001^§^
Primary	449 (22.2)	381 (21.2)	38 (29.5)	21 (30.0)	9 (37.5)	
Middle, secondary, or matric	458 (22.6)	432 (24.0)	22 (17.1)	3 (4.3)	1 (4.2)	
Higher secondary or more	120 (5.9)	118 (6.6)	1 (0.8)	1 (1.4)	0 (0.0)	
Missing	1	1	0	0	0	
Occupational status						
Farmer or agricultural laborer	113 (5.6)	98 (5.4)	8 (6.2)	5 (7.3)	2 (8.3)	0.066^§^
Other employment or trade	413 (20.4)	370 (20.6)	28 (21.7)	10 (14.5)	5 (20.8)	
Housewife	592 (29.3)	550 (30.6)	25 (19.4)	14 (20.3)	3 (12.5)	
Student	680 (33.6)	595 (33.1)	47 (36.4)	28 (40.6)	10 (41.7)	
Child, not in school	175 (8.7)	146 (8.1)	17 (13.2)	8 (11.6)	4 (16.7)	
None	49 (2.4)	41 (2.3)	4 (3.1)	4 (5.8)	0 (0.0)	
Missing	2	1	0	1	0	
Number of people in household						
Mean (SD, range)	4.8 (2.0, 1–20)	4.8 (2.0, 1–20)	4.5 (1.5, 2–12)	4.3 (1.9, 2–11)	5.3 (2.3, 2–11)	0.054^‡^
Fever of 99.5°F or higher						
No	1,483 (73.5)	1,382 (77.0)	35 (27.1)	58 (82.9)	8 (33.3)	<0.001
Yes	534 (26.5)	412 (23.0)	94 (72.9)	12 (17.1)	16 (66.7)	
Missing	7	7	0	0	0	
Asymptomatic infection						
No	122 (54.7)	–	94 (72.9)	12 (17.1)	16 (66.7)	<0.001
Yes	101 (45.3)	–	35 (27.1)	58 (82.9)	8 (33.3)	

MC, malaria camp; *P. falciparum, Plasmodium falciparum*; PCR, polymerase chain reaction; *pfhrp2*, *Plasmodium falciparum* histidine-rich protein 2; RDT, rapid diagnostic test.

* Missing/inconclusive category includes RDT–/PCR+ *Plasmodium falciparum* infections that were either not tested for *pfhrp2* deletion or had inconclusive *pfhrp2* deletion results, as well as *Plasmodium vivax* mono-infections.

^†^ *P*-value calculated using Pearson’s χ^2^ test, unless otherwise noted.

^‡^ *P*-value calculated using analysis of variance.

^§^ *P*-value calculated using Fisher’s exact test.

The crude and adjusted multilevel, multinomial logistic regression models are presented in Table 5. Participants who received the MC intervention (arm A) had a lower risk of being infected with *P. falciparum* parasites containing a *pfhrp2* gene deletion relative to no infection when compared with participants who did not receive the intervention (arm B; aRRR = 0.3; 95% CI = 0.1–1.0). Participants who received the MC intervention also had a lower risk of being infected with *P. falciparum* parasites with an intact *pfhrp2* gene relative to no infection when compared with participants who did not receive the intervention (aRRR = 0.4; 95% CI = 0.2–1.1).

**Table 5:** Multilevel, multinomial logistic regression models estimating the association between the malaria camp intervention and *Plasmodium falciparum* infections with and without *pfhrp2* gene deletions (*n* = 2,024)

	*P. falciparum* With *pfhrp2* Gene Deletion vs. No Infection		*P. falciparum* With Intact *pfhrp2* Gene vs. No Infection		*P. falciparum* With *pfhrp2* Gene Deletion vs. *P. falciparum* With Intact *pfhrp2* Gene	
	RRR (95% CI)	aRRR (95% CI)	RRR (95% CI)	aRRR (95% CI)	RRR (95% CI)	aRRR (95% CI)
*P. falciparum* infection at baseline						
Arm B (delayed MC)	Ref	Ref	Ref	Ref	Ref	Ref
Arm A (new MC)	7.9 (1.6–38.6)*	8.2 (1.7–39.0)*	3.7 (0.7–18.2)	3.7 (0.8–18.1)	1.4 (0.1–23.5)	1.4 (0.1–23.2)
*P. falciparum* infection by study visit						
Baseline	Ref	Ref	Ref	Ref	Ref	Ref
Follow-up 3	0.2 (0.1–0.5)*	0.2 (0.1–0.5)*	0.8 (0.4–1.6)	0.8 (0.5–1.6)	0.2 (0.1–0.6)*	0.2 (0.1–0.6)*
*P. falciparum* infection at follow-up 3 by study arm						
Arm B (delayed MC)	Ref	Ref	Ref	Ref	Ref	Ref
Arm A (new MC)	0.3 (0.1–1.0)*	0.3 (0.1–1.0)^†^	0.4 (0.2–1.0)^†^	0.4 (0.2–1.1)^†^	0.7 (0.1–3.7)	0.7 (0.1–3.7)
Age						
(in years)		1.0 (1.0–1.0)*		1.0 (1.0–1.0)*		1.0 (1.0–1.0)
Sex						
Male		Ref		Ref		Ref
Female		0.7 (0.5–1.1)		1.0 (0.6–1.6)		0.7 (0.3–1.3)

aRRR, adjusted relative risk ratio; MC, malaria camp; *P. falciparum, Plasmodium falciparum*; *pfhrp2*, *Plasmodium falciparum* histidine-rich protein 2; Ref, reference category; RRR, relative risk ratio.

* *P*-value <0.05.

^†^ *P*-value <0.1.

## DISCUSSION

Studies characterizing *pfhrp2* gene deletions have gained importance over the last decade because of the increasing failure of PfHRP2-based RDTs used in malaria-endemic areas around the globe.^17^^–^^19^^,^^39^ In this study, we report that 61.6% of subpatent infections identified in 15 study villages across two districts of Odisha, India, harbored *pfhrp2* gene deletions (96.2% of all deletions) within the exon 2 region. These RDT failures can be confidently attributed to the presence of *pfhrp2* exon 2 deletions and not the RDT limit of antigen detection or low parasitemia infections, given that 82.6% of the *pfhrp2* gene deletions were identified in febrile individuals (i.e., cases of clinical malaria).

PfHRP2-based RDTs remain highly sensitive in detecting *P. falciparum* infections in some settings, with a recent study from central India reporting a sensitivity of 95.3% among symptomatic patients.^40^ Pati et al. reported PfHRP2-targeted RDT failure in 15% (58 of 384) of *P. falciparum* infections from Odisha State between 2013 and 2016, with 65.5% (*n* = 38) of failures due to deletions in *pfhrp2*.^28^ A second report from Odisha detected only 18 false negative RDT results out of 229 (7.9% RDT failure) *P. falciparum*-positive samples collected in 2014, with 66% (*n* = 12) due to deletions in *pfhrp2*.^27^ However, the samples tested in these previous studies were collected from suspected malaria patients; our study is the first to examine *pfhrp2* deletions among both symptomatic and asymptomatic individuals in Odisha. Our findings demonstrate a marked increase in PfHRP2-based RDT failure in the region (258 of 324 total *P. falciparum* infections; 79.6% RDT-negative), with a similar proportion of failures due to the presence of *pfhrp2* deletions (61.6% versus 65.5%^28^ and 66%^27^). Altogether, this should be addressed if continued progress in both case management and transmission interruption is to be attained.

The WHO recommends that false-negative PfHRP2-detecting RDT results should be investigated for *pfhrp2* and *pfhrp3* gene deletions and that representative surveys or population-based surveillance should be used to determine prevalence in regions in which *pfhrp2* deletions are present.^41^^–^^43^ If the prevalence of false-negative RDT infections with *pfhrp2* deletions is higher than 5% of total *P. falciparum* infections in a region, the WHO recommendations state that the use of PfHRP2-detecting RDTs should be discontinued and alternative diagnostic methods should be adopted.^41^ Although false negative results pose a grave concern when using PfHRP2-based RDTs, false positive results due to the persistence of PfHRP2 protein, even after the clearance of the parasite, are not unknown either.^44^ However, RDTs are often the only feasible diagnostic test in many locations, and options for RDTs that diagnose *P. falciparum* infection without detecting PfHRP2 are extremely limited.^15^^,^^21^^,^^45^ Diagnostic testing is a crucial component of malaria elimination strategies, and nearly all *P. falciparum* RDTs are designed to identify PfHRP2. The findings of this study highlight the urgent need to develop new RDTs that use alternative targets (e.g., *Plasmodium falciparum* Glutamate Dehydrogenase^46^ and *Plasmodium falciparum* Glutamic acid rich protein^47^) to diagnose *P. falciparum* infections.

In our study region, RDTs are the most feasible tool for identifying *Plasmodium* infections, and the Odisha State malaria control program relies on PfHRP2-detecting RDTs to detect malaria cases caused by *P. falciparum* infections. Our evaluation of the DAMaN MC intervention found that mass screening and treatment, combined with additional components (i.e., community programming and comprehensive vector control measures, including long-lasting insecticidal net distribution and indoor residual spraying), reduced the relative risk of infection for both *P. falciparum* with *pfhrp2* deletions (aRRR = 0.3; 95% CI = 0.1–1.0) and *P. falciparum* with intact *pfhrp2* genes (aRRR = 0.4; 95% CI = 0.2–1.1) when compared with the use of mass screening with RDTs alone. Although this analysis examined a secondary outcome and was not powered for statistical significance, the effect estimates and confidence intervals indicate likely reductions in relative risk (*P*-values <0.1). Given that both intervention arms received mass testing with PfHRP2-detecting RDTs, this potential reduction may be related to individual intervention components other than testing, or the combined effect of the package of interventions. These results indicate that testing with PfHRP2-detecting RDTs may not be sufficient in reducing the transmission of *Plasmodium* infections in locations with *pfhrp2* deletions; however, they also indicate that the MC intervention may be useful in targeting *P. falciparum* infections with *pfhrp2* deletions when alternative diagnostic methods are not available.

To assess the patterns and locations of *pfhrp2* gene deletions, we targeted multiple genetic regions, including exon 1–2, exon 2, the upstream flanking gene, and the downstream flanking gene. We identified the greatest proportion of deletions in the exon 2 region (96.2%). Our findings are similar to those of a study conducted in Ghana that reported a high proportion of deletions (39.5%) in the exon 2 region^48^ but in contrast to a study conducted in Ethiopia that reported the lowest proportion of deletions in the exon 2 region (17.9%).^13^ Another study conducted on Brazilian samples found the highest proportion of deletions in the upstream flanking gene (21.2%), followed by the exon 2 region (13.6%) and the downstream flanking gene (0.5%), with no deletions in the exon 1–2 region.^49^ Thus, it appears that deletions in the *pfhrp2* gene and its flanking genes have diversified abundance in malarious regions across the globe. Further, their individual contributions to RDT failure remain to be fully understood.

We identified 13 unique arrangements of 10 different repeat motifs in 17 RDT– *P. falciparum*-confirmed samples (repeat types 1–8, 12, 19). Our results align with a previous report from India that identified all the repeat types mentioned, along with four additional types (i.e., repeat types 9, 10, 13, and 15).^27^ The high diversity in the arrangement of amino acids and detection of many unique sequences was highlighted in a study from Kenya, wherein 94% of the samples analyzed had unique sequences.^38^ It is worth noting, however, that reports from other settings, such as Peru, found minimal diversity among their amino acid sequences.^10^ All the protein sequences in the RDT–/PCR+ samples started with a type 1 and ended with a type 12 motif, which is the most common pattern observed from various geographical locations.^38^^,^^50^ However, a report from Senegal suggested that the occurrence of repeat type 12 was quite uncommon. We also detected seven variants in six of the amino acid repeats with one to two amino acid substitutions, five of which are novel variants, whereas the other two have been reported previously.^38^ The variants detected in the RDT– samples were not found in the RDT+ samples (Table 3), suggesting that the presence of such novel variants in the deletion-prone exon 2 region of the *pfhrp2* gene could be associated with selection for de novo gene deletion. Recent studies from Djibouti^52^ and Eritrea^53^ suggest that *pfhrp2* deletions may be arising de novo or emerging independently and have been expanding in the Horn of Africa.

The prediction of RDT sensitivity using a product of the number of type 2 and type 7 repeats was initially suggested by Baker and colleagues in samples with a parasite density of <250 parasites per µl.^12^ However, we were not able to detect an association between RDT sensitivity and the presence of type 2 and type 7 repeats in our samples (Supplemental Table 1). Instead, the false negative RDT results in samples without a *pfhrp2* gene deletion from our study may have been a consequence of plasma PfHRP2 antigen levels below the limit of detection (Supplemental Figure 2A). This observation is in line with a recent study from central Vietnam, wherein false-negative RDT results have been attributed to low PfHRP2 plasma levels.^54^

Our study was limited to patient isolates collected from 15 study villages across two districts of Odisha State. Given the many inherent sampling limitations in field settings, we support a large-scale mapping across all malaria-endemic districts to generate a complete understanding of *P. falciparum* with *pfhrp2* gene deletions circulating in the state. Just over one-third (38.4%) of RDT-negative/PCR-positive *P. falciparum* infections identified in our study did not harbor *pfhrp2* deletions. These infections are most likely attributable to peripheral parasitemias below the limit of RDT detection or low PfHRP2 antigen levels in the peripheral plasma (Supplemental Figure 2A–B).

## CONCLUSION

Our study infers that a sizeable proportion (61.6%) of the subpatent *P. falciparum* infections from two districts in the highly endemic state of Odisha, India probably evaded routine detection by PfHRP2-based RDT kits because of deletions in the targeted *pfhrp2* gene. The majority (96.2%) of the deletions were detected in the exon 2 locus and in symptomatic, febrile individuals (82.6%). In addition, we determined the novel variants of the amino acid repeat motifs (231–293 amino acids) detected at the *pfhrp2* exon 2 locus in a subset of subpatent infections (*n* = 17) with an intact *pfhrp2* gene differed from the variants of RDT+ clinical control samples (*n* = 10; Table 3). One hypothesis is that the observed differences may be associated with suboptimal production of PfHRP2 in the subpatent infection samples (Supplemental Figure 2A), resulting in novel variants in the deletion-prone (exon 2 region) *pfhrp2* gene rendering the selection for de novo gene deletion in the PfHRP2 RDT– samples over time. Although this has been observed in distant, geographically distinct settings,^51^^,^^52^ this hypothesis requires further investigation. RDTs targeting PfHRP2 remain a widely used diagnostic tool, leaving opportunities for *P. falciparum* infections with *pfhrp2* deletions to go undiagnosed, particularly in remote field settings in which microscopy and molecular methods are unavailable. Undiagnosed infections allow *P. falciparum* with *pfhrp2* gene deletions to go untreated and serve as a reservoir for infection, enabling parasites with the deletion to be further transmitted. The results of this study indicate that testing with RDTs may not be sufficient in reducing the transmission of *P. falciparum* infections in regions with *pfhrp2* deletions as a sole intervention; however, comprehensive interventions that supplement testing with vector control and education strategies were effective in reducing the prevalence of infections both with and without the gene deletion. Interventions that use mass testing using PfHRP2-detecting RDTs should be accompanied by comprehensive intervention strategies to prevent the transmission of *P. falciparum* infections with *pfhrp2* deletions.

## Supplemental Materials

10.4269/ajtmh.24-0330 Supplemental Materials
