# Supplementary material for: Genetic Characterization of Plasmodium falciparum Histidine-Rich Protein 2 Deletions and Their Impact on Malaria Interventions in Odisha, India
Source: Am J Trop Med Hyg. 2024 Dec 17;112(3):601–9. doi: 10.4269/ajtmh.24-0330 (PMC11884295; doi:10.4269/ajtmh.24-0330)
Supplement: Supplemental Materials [file tpmd240330.SD1.pdf]

SUPPLEMENTARY DATA

Suppl. Table S1. Prediction of RDT sensitivity using the multiplication product of Type 2 and Type 7 Repeats

Group	No. of RDT-/ssPCR+ Samples (%)	No. of RDT+/ssPCR+ Samples (% frequency)
**Group A**	1 (5.9)	0 (0)
**Group B**	10 (58.8)	3 (30)
**Group I**	2 (11.8)	2 (20)
**Group C**	4 (23.5)	5 (50)

Group A: Very sensitive (Type 2 X Type 7>100); Group B: Sensitive (Type 2 X Type 7= 50-100); Group I: Borderline ((Type 2 X Type 7= 44-49); Group C: Not sensitive (Type 2 X Type 7< 43)

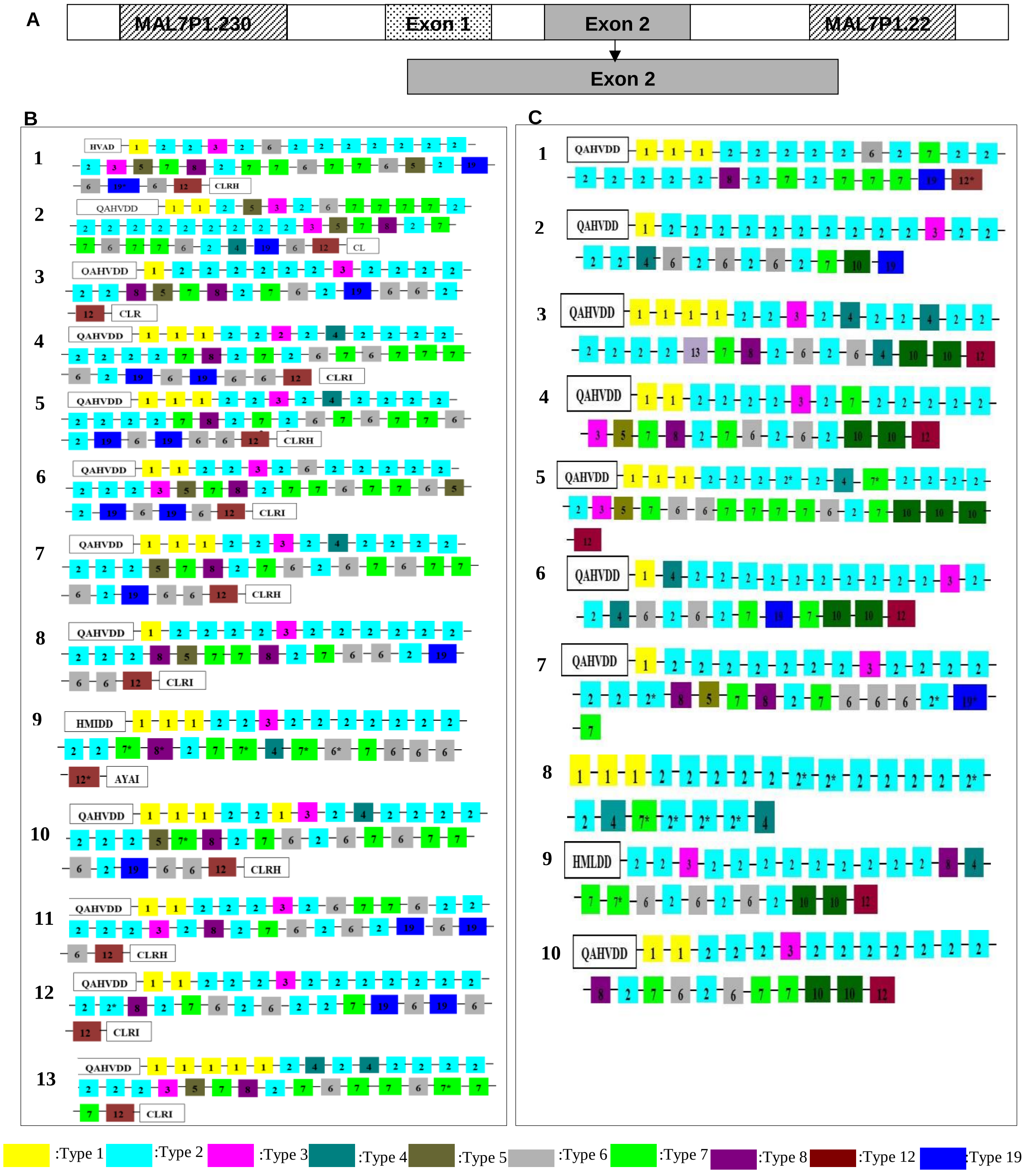

Suppl. Figure S1. Schematic of *pfhrp2* gene and its upstream and downstream flanking regions **(A)** and PfHRP2 exon 2 protein features of RDT-/ ssPCR+ samples **(B)** and RDT+/ssPCR+ samples **(C)**. Each amino acid repeat type (Baker *et al*, 2005; Nderu *et al*, 2019) is indicated by colour block. All samples began with a type 1 repeat, and eight ended with a type 12 repeat. Four repeat types that differed from type 2 (B.12), type 7 (B.9,10, 13), type 8 (B.9), and type 19 (B.1) by a single amino acid were also detected among the isolates. A variant of repeat type 12 with amino acid substitutions at two positions was also detected in one (B.9) sample. Among the clinical malaria control group (PfHRP2 RDT+) samples, the HRP2 protein consisted of 187-274 amino acids. Like the RDT- PCR+ samples, repeat types 1-7, 12 and 19 were detected in the clinical control sample set with two additional repeat types, type 10 and type 13. Among the clinical control samples, type 2 was the most abundantly occurring repeat type. The variants of repeat types 2 (C.5,7,8), 7 (C.5,8,9), and 19 (C.7) were detected in this set of samples, with repeat type 19 being novel in nature. Among the variants detected, there was only one common variant between the RDT- and RDT+ sample sets, a variant of type 2 repeat.

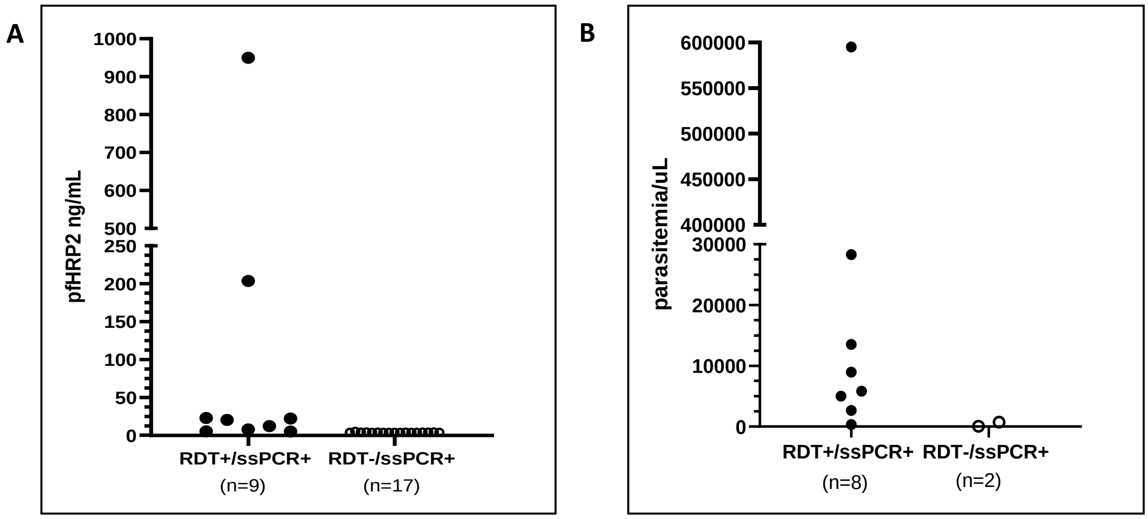

Supplemental Figure S2. (A) Plasma PfHRP2 levels and **(B)** Peripheral parasitemia, in RDT+/ssPCR+ vs. RDT-/ssPCR+ sample subsets
